# LIFEBIO: PARTICIPANTS' EXPERIENCES IN A TELEPHONIC-BASED REMINISCENCE PROGRAM

**DOI:** 10.1093/geroni/igz038.658

**Published:** 2019-11-08

**Authors:** Janella Hudson, Rifky Tkatch, Karen Keown, Michael McGinn, Ellen Wicker

**Affiliations:** 1 Optum, Ann Arbor, Michigan, United States; 2 UnitedHealthcare, Minneapolis, Minnesota, United States; 3 AARP Services, Inc., Washington, District of Columbia, United States

## Abstract

Older adults facing age-related transitions are at increased risk for depression and loneliness. Reminiscence therapy has demonstrated positive outcomes for older adults, including improved socialization and reduced depression. A program known as LifeBio was designed as a group intervention to engage participants by capturing their life stories to positively impact wellness through reminiscence. This program was adapted to a telephonic format for the Aging Strong 2020 initiative. Semi-structured interviews eliciting feedback about participants’ experiences in the program were conducted with 24 participants. Respondent feedback indicated that those with limited mobility and fewer social connections reported the greatest benefit. Many identified the value of journaling as a form of recordkeeping for family members, but reported varying degrees of journal completion. Suggestions for improvement included future facilitation of two-way communication with other participants and opportunities for face-to-face interaction in group settings.

